# On the aetiology of autism

**DOI:** 10.1111/j.1651-2227.2010.01883.x

**Published:** 2010-08

**Authors:** John J Cannell

**Affiliations:** Atascadero State Hospital – PsychiatryAtascadero, CA, USA


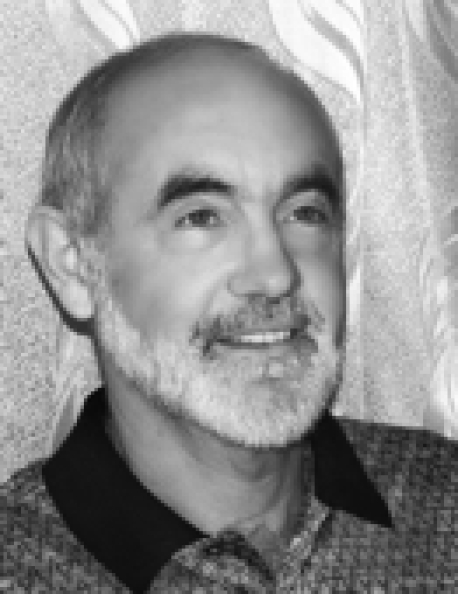


I have suggested that the primary environmental trigger for autism is not vaccinations, toxins or infections, but gestational and early childhood vitamin D deficiency (1,2). Subsequently, the title of an article in *Scientific American* recently asked, ‘What if vitamin D deficiency is a cause of autism?’ (3) Since then, an article on vitamin D and autism in *Acta Paediatrica* (4) and the accompanying commentary (5) have added to the accelerating suspicion that vitamin D deficiency – either during pregnancy or early childhood – may be an environmental trigger for the genetic disease of autism.

I will not repeat the evidence from my original 2007 paper and I am well aware that evidence consistent with a theory is not proof of that theory. Instead, I concentrate on papers either published since my paper, or on evidence I overlooked when researching the original paper. Finally, I explain my reasons why this theory deserves immediate attempts to disprove it.

Simultaneous with the above two *Acta Paediatrica* publications, a paper elsewhere reported that among 117 adult psychiatric outpatients is Sweden with various diagnoses, the 10 patients with autism had the lowest vitamin D levels of any of the groups studied, a mean of 12 ng/mL (31 nM), approaching the osteomalacic range for adults and the rachitic range for children (6). Even more interesting, some of the patients seemed to improve when treated with an average dose of about 4000 IU of vitamin D/day. The authors did not say if the improvement occurred in the autism group.

A 2008 review detailed the devastating effect gestational vitamin D deficiency has on developing mammalian brains (7). Unfortunately, the tiny 10 μg (400 IU) dose in prenatal vitamins is virtually irrelevant in preventing the current epidemic of gestational vitamin D deficiency (8). For this reason, in 2007, the Canadian Paediatric Society cautioned pregnant women they may require not 400 IU/day but 2000 IU/day, or more, to prevent gestational vitamin D deficiency (9).

If adequate amounts of vitamin D prevent autism, one would expect children with rickets to have an increased risk of autism. To my knowledge, the neuropsychiatric symptoms of rickets have not been studied in the modern era. However, at least two old papers have addressed it (10,11), both published before Kanner described autism in 1943. Both papers describe ‘weak mindedness,’‘feeble minds,’‘mental dullness,’ unresponsiveness and developmental delays. Even more intriguing, both papers report that the mental condition in rickets improved with vitamin D.

Another of the mysteries of autism is the apparent increased incidence of autism in the children of richer college-educated parents, especially women, a finding announced a few months ago (12). Actually, this is not a new finding. As I discussed in my original paper, this has been known since the early 1980s but was dismissed as being because of ascertainment bias. This very recent report correlates well with a 2007 CDC report (13), which found a similar increased risk for the wealthy and well-educated, findings the authors tried but could not dismiss as being because of ascertainment bias. If the vitamin D theory is true, autism should be more common in richer well-educated mothers, who are more likely than other mothers to practice sun avoidance and use sunblock (1,2).

In his invited commentary, Dr. Eyles asked, ‘Does skin colour modify the risk’ of autism, noting melanin in the skin is an effective sunblock. Such studies are difficult as they raise sensitive social issues. Nevertheless, three of four recent U.S. studies found a higher incidence of autism in black children, sometimes appreciably higher (1,2). As Fernell et al. report, the Somali immigrants in Sweden call autism the ‘Swedish disease’ and Somali immigrants in Minnesota call it the ‘American disease,’ but in equatorial Somalia, autism has no name.

Toxins delivered by water or air pollution appear to damage the genome of the vitamin D deficient (14). If exposure to such toxins were the main contributors to autism incidence, then we should have seen an autism incidence pattern in the US that mirrored air and water pollution with a dramatic increase in the 1950s, a peak in the 1960s and then a progressive decline by the early 1980s, coincident with enactment and enforcement of the US clean air and clean water acts of the 1960s.

Another possibility is that air pollution from Eastern Europe, India and China, which has been increasing in the last 20 years, has engendered the current crop of autism. However, why would foreign air pollution of today do what American air pollution of the 1950s and 60s could not?

Another paper published after my original paper found that autistic boys have unexplained reductions in metacarpal bone thickness (15). At some time in their life, these children laid down less cortical bone than normal children, a finding consistent with undetected and untreated childhood or even intrauterine vitamin D deficiency.

Yet another recent paper reported that the prevalence of autism in three U.S. states was higher in areas of higher precipitation and clouds (16). The 2005 autism prevalence rate among school-aged children, after controlling for differences in population size, demographic characteristics, per capita income and state, was higher in cloudy areas. The association of autism prevalence and the mean annual precipitation received by a county between 1987 and 2001 was positive and significant (p = 0.0034; 95% confidence interval, 0.0018–0.0050). Clouds and rain retard vitamin D-producing ultraviolet B light from penetrating the atmosphere.

Surprisingly, high maternal seafood consumption, of the type known to be contaminated with mercury, has been associated with fewer – not more – autistic markers in the offspring (17). Lower maternal seafood intake during pregnancy was associated with low verbal intelligence quotient, suboptimum outcomes for prosocial behaviour, fine motor, communication and social development scores. While the omega-3 and mercury content of fish is well known, less well known is the fact that fish is one of the few foods with significant amounts of vitamin D, which, as referenced earlier, protects the genome from damage by toxins.

While the urban/rural gradient in rickets is well known and was one of the keys to discovering that sunlight prevented rickets, less well known is a meta-analysis showing a twofold urban/rural gradient for autism (18). Similar to rickets, city life affords less vitamin D, because of tall buildings, indoor occupations and increased urban air pollution, all of which block ultraviolet B light from penetrating the atmosphere.

Finally, a 2008 paper reported that autism was more common among mothers who took antiepileptic drugs (19). A comment to the authors (20) detailed the evidence that antiepileptic drugs are one of the few classes of drugs that consistently and significantly interfere with vitamin D metabolism, lowering 25(OH)D levels.

I agree with Dr Eyles’ comment that the vitamin D theory is ‘highly parsimonious.’ Indeed, it was love of parsimony that led me to first hypothesize that vitamin D is intimately involved in the pathology and epidemiology of both autism and influenza (21). Some have speculated that the excess of winter births in autism is explained by wintertime maternal viral infections. Obviously, another, and more parsimonious explanation, is that vitamin D is involved in all three.

The vitamin D theory of autism does not diminish genetic contributions to autism occurrence. Indeed, without the genetic tendency for autism, I suspect that severe maternal or early childhood vitamin D deficiency may cause bone abnormalities, as referenced above, with no evidence autism. All that the current epidemic of maternal and early childhood vitamin D deficiency does, with its resultant neural deficiency in the pluripotent neurosteroid calcitriol, is to allow the genetic tendency for autism to express itself.

If this theory is true, the path towards effective prevention – and perhaps a treatment effect if adequate physiological doses of vitamin D are given – is so simple, so safe, so inexpensive, so readily available and so easy, that it defies imagination. Seventeen vitamin D experts recently stated, ‘In our opinion, children with chronic illnesses such as autism, diabetes and/or frequent infections should be supplemented with higher doses of sunshine or vitamin D3, doses adequate to maintain their 25(OH)D levels in the mid-normal of the reference range [65 ng/mL (162 nmol/L)] – and should be so supplemented year round.’ (22)

Finally, if true, a darker side of the theory emerges. To some real but unknown extent, autism is an iatrogenic disease, caused by governments, organizations, committees, newspapers and physicians who promulgated the current warnings about sun-exposure for pregnant women and young children without any understanding of the tragedy they engendered.
